# (11*R*)-13-[2-(4-Hydroxy­phen­yl)ethyl­amino]-4,5-ep­oxy-11,13-dihydro­costunolide monohydrate

**DOI:** 10.1107/S1600536808003863

**Published:** 2008-02-29

**Authors:** Shama. Nasim, Sean Parkin, Peter A. Crooks

**Affiliations:** a Department of Pharmaceutical Sciences, College of Pharmacy, University of Kentucky, Lexington, KY 40536, USA; b Department of Chemistry, University of Kentucky, Lexington, KY 40506, USA

## Abstract

The title compound (systematic name: 12-{[2-(4-hydroxyphenyl)ethyl]aminomethyl}-4,8-dimethyl-3,14-dioxatricyclo[9.3.0.0^2,4^]tetradec-7-en-13-one monohydrate), C_23_H_31_NO_4_·H_2_O, was obtained by the reaction of tyramine with parthenolide. The configuration of the new chiral center in the title compound is *R*, establishing the stereospecificity of the amination reaction. The water molecule is disordered over three positions; the site occupancy factors are 0.45, 0.40 and 0.15.

## Related literature

For related literature, see: Allen *et al.* (1987 ▶); Crooks *et al.* (2005 ▶); Desiraju & Steiner (1999 ▶); Hewlett *et al.* (1996 ▶); Nasim *et al.* (2007*a* ▶,*b* ▶).
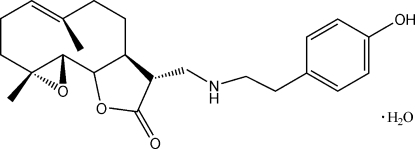


## Experimental

### 

#### Crystal data

C_23_H_31_NO_4_·H_2_O
*M* *_r_* = 403.50
Monoclinic, 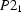

*a* = 10.8307 (7) Å
*b* = 6.9478 (5) Å
*c* = 14.4835 (9) Å
β = 94.631 (3)°
*V* = 1086.32 (12) Å^3^
*Z* = 2
Cu *K*α radiation
μ = 0.70 mm^−1^
*T* = 90.0 (2) K
0.25 × 0.10 × 0.03 mm

#### Data collection

Bruker X8 Proteum diffractometer
Absorption correction: multi-scan (*SADABS* in *APEX2*; ­Bruker–Nonius, 2004 ▶) *T*_min_ = 0.763, *T*_max_ = 0.979
13335 measured reflections
3532 independent reflections
3420 reflections with *I* > 2σ(*I*)
*R*_int_ = 0.031

#### Refinement

*R*[*F* ^2^ > 2σ(*F* ^2^)] = 0.041
*wR*(*F* ^2^) = 0.113
*S* = 1.07
3532 reflections
276 parameters
7 restraints
H atoms treated by a mixture of independent and constrained refinement
Δρ_max_ = 0.40 e Å^−3^
Δρ_min_ = −0.20 e Å^−3^
Absolute structure: Flack (1983),  1365 Friedel pairs
Flack parameter: 0.12 (6)

### 

Data collection: *APEX2* (Bruker–Nonius, 2004 ▶); cell refinement: *APEX2*; data reduction: *APEX2*; program(s) used to solve structure: *SHELXS97* (Sheldrick, 2008 ▶); program(s) used to refine structure: *SHELXL97* (Sheldrick, 2008 ▶); molecular graphics: *XP* in *SHELXTL* (Sheldrick, 2008 ▶); software used to prepare material for publication: *SHELXL97* and local procedures.

## Supplementary Material

Crystal structure: contains datablocks global, I. DOI: 10.1107/S1600536808003863/hg2375sup1.cif

Structure factors: contains datablocks I. DOI: 10.1107/S1600536808003863/hg2375Isup2.hkl

Additional supplementary materials:  crystallographic information; 3D view; checkCIF report

## Figures and Tables

**Table 1:** Hydrogen-bond geometry (Å, °)

*D*—H⋯*A*	*D*—H	H⋯*A*	*D*⋯*A*	*D*—H⋯*A*
N1—H1*N*⋯O3	0.94 (3)	2.22 (3)	2.979 (3)	137 (2)
O1′—H1′⋯O1*W*1^i^	0.84	1.75	2.568 (4)	164
O1′—H1′⋯O1*W*2^i^	0.84	2.01	2.832 (6)	166

Symmetry code: (i) 
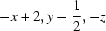
.

**Fig. 1.**
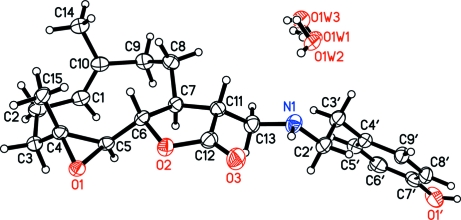
A view of the asymmetric unit of the title compound, with displacement ellipsoids drawn at the 50% probability level; H atoms are shown as small spheres of arbitrary radii.

**Fig. 2.**
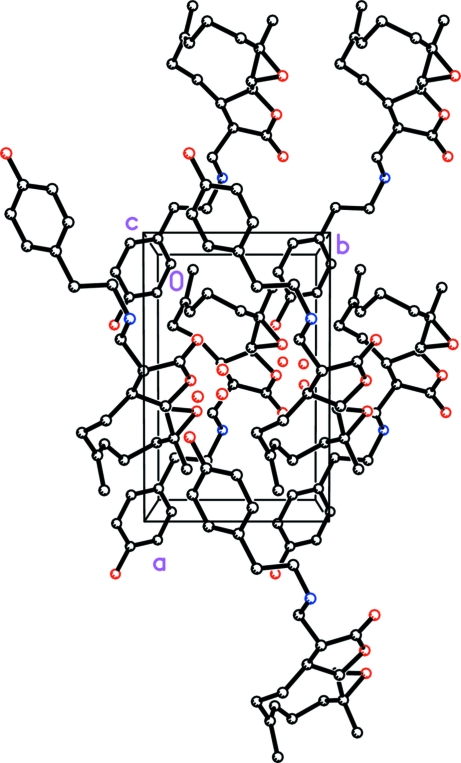
A packing diagram, viewed down the a axis; hydrogen atoms have been omitted for clarity.

## supplementary crystallographic information

### Comment

Parthenolide, is a germacrene sesquiterpene lactone. It has been isolated from several different species of plant in the Asteraceae (Compositae) family, feverfew (Tanacetum parthenium) being one of them. (Hewlett *et al.*, 1996). The title compound was synthesized as part of an ongoing drug discovery effort (Crooks *et al.*, 2005)and is the adduct of the neurotransmiter, tyramine and the cytotoxic sesquiterpene, parthenolide. This compound was found to crystallize as the monohydrate, in contrast to other structurally related parthenolide analogs (Nasim *et al.*, 2007*a*, 2007*b*). The side-chain was found to be in a fully extented conformation. The absolute stereochemistry of the newly formed methine carbon at C-11 was found to be *R*, which is typical of such amine adducts of parthenolide (Nasim *et al.*, 2007*a*, 2007*b*). Bond distances and angles within the molecule were quite regular with average normal bond lengths (Allen *et al.*, 1987). A weak hydrogen bond (Desiraju *et al.*, 1999) is observed between N-1H and O3 of the carbonyl oxygen of the 5-membered lactone ring (2.22 (3) A°, 2.97 (3) A°, 137.0 (2) °) (Table 1),

### Experimental

The title compound was prepared utilizing the general procedure reported earlier (Nasim *et al.*, 2007*a*, 2007*b*). Anal. (C_23_H_31_NO_4_. H_2_O): C 68.46, H 8.24, N 3.47%. Found C 68.20, H 7.94, N 3.41%.

### Crystal data

**Table:**

C_23_H_31_NO_4_·H_2_O_1_	*F*_000_ = 436
*M**_r_* = 403.50	*D*_x_ = 1.234 Mg m^−^^3^
Monoclinic, *P*2_1_	Cu *K*α radiation λ = 1.54178 Å
Hall symbol: P 2yb	Cell parameters from 5637 reflections
*a* = 10.8307 (7) Å	θ = 3.1–68.0º
*b* = 6.9478 (5) Å	µ = 0.70 mm^−^^1^
*c* = 14.4835 (9) Å	*T* = 90.0 (2) K
β = 94.631 (3)º	Lath, colourless
*V* = 1086.32 (12) Å^3^	0.25 × 0.10 × 0.03 mm
*Z* = 2	

### Data collection

**Table:**

Bruker X8 Proteum diffractometer	3532 independent reflections
Radiation source: fine-focus rotating anode	3420 reflections with *I* > 2σ(*I*)
Monochromator: Bruker Helios multilayer optics	*R*_int_ = 0.031
Detector resolution: 18 pixels mm^-1^	θ_max_ = 68.1º
*T* = 90.0(2) K	θ_min_ = 3.1º
ω and φ scans	*h* = −12→12
Absorption correction: multi-scan(SADABS in APEX2; Bruker–Nonius, 2004)	*k* = −8→5
*T*_min_ = 0.763, *T*_max_ = 0.979	*l* = −17→17
13335 measured reflections	

### Refinement

**Table:**

Refinement on *F*^2^	Hydrogen site location: inferred from neighbouring sites
Least-squares matrix: full	H atoms treated by a mixture of independent and constrained refinement
*R*[*F*^2^ > 2σ(*F*^2^)] = 0.041	*w* = 1/[σ^2^(*F*_o_^2^) + (0.0695*P*)^2^ + 0.347*P*] where *P* = (*F*_o_^2^ + 2*F*_c_^2^)/3
*wR*(*F*^2^) = 0.113	(Δ/σ)_max_ = 0.009
*S* = 1.07	Δρ_max_ = 0.40 e Å^−^^3^
3532 reflections	Δρ_min_ = −0.20 e Å^−^^3^
276 parameters	Extinction correction: none
7 restraints	Absolute structure: Flack (1983), 1365 Friedel pairs
Primary atom site location: structure-invariant direct methods	Flack parameter: 0.12 (6)
Secondary atom site location: difference Fourier map	

### Special details

**Table:**

Geometry. All e.s.d.'s (except the e.s.d. in the dihedral angle between two l.s. planes) are estimated using the full covariance matrix. The cell e.s.d.'s are taken into account individually in the estimation of e.s.d.'s in distances, angles and torsion angles; correlations between e.s.d.'s in cell parameters are only used when they are defined by crystal symmetry. An approximate (isotropic) treatment of cell e.s.d.'s is used for estimating e.s.d.'s involving l.s. planes.
Refinement. Refinement of *F*^2^ against ALL reflections. The weighted *R*-factor *wR* and goodness of fit *S* are based on *F*^2^, conventional *R*-factors *R* are based on *F*, with *F* set to zero for negative *F*^2^. The threshold expression of *F*^2^ > σ(*F*^2^) is used only for calculating *R*-factors(gt) *etc*. and is not relevant to the choice of reflections for refinement. *R*-factors based on *F*^2^ are statistically about twice as large as those based on *F*, and *R*- factors based on ALL data will be even larger.CIFCHECK squawks about H atoms being detached from their oxygen atom in the third disorder component of the water. They are are in fact 0.85Å from O1W3, but the occupancy factor is only 0.15. They were included so as to get the atom count correct, and no claim is made for their veracity.This three-component disorder for the water is consistent with the fact that the crystals appeared to be cracking due to solvent loss. The actual crystal chosen was carefully cut so that it did not have any cracks, but still it is quite likely that the three-part disorder is related to this observed tendency for these crystals to dry and crack._publ_section_exptl_refinement: H atoms were found in difference Fourier maps and subsequently placed in idealized positions with constrained C—H distances of 0.95 Å (C_sp2_—H), 1.00 Å (*R*_3_CH), 0.99 Å (*R*_2_CH_2_), 0.98 Å (RCH_3_), 0.84 Å (OH) and 0.85 Å (OH_2_) except for the NH hydrogen coordinates, which were refined. Hydrogen atom *U*_iso_(H) values were set to 1.2*U*_eq_ or 1.5*U*_eq_ (RCH_3_, OH, OH_2_) of the attached atom. Since the water molecule was severely disordered the hydrogen atoms were placed in reasonable but not necessarily correct positions, and were subsequently fixed.

### Fractional atomic coordinates and isotropic or equivalent isotropic displacement parameters (Å^2^)

**Table:**

	*x*	*y*	*z*	*U*_iso_*/*U*_eq_	Occ. (<1)
O1	0.37395 (12)	0.7648 (2)	0.49755 (9)	0.0254 (3)	
O2	0.49033 (12)	0.7364 (2)	0.33035 (10)	0.0271 (3)	
O3	0.63676 (15)	0.7794 (3)	0.23432 (11)	0.0412 (4)	
C1	0.25041 (19)	0.2241 (3)	0.48640 (15)	0.0280 (4)	
H1	0.3290	0.1714	0.5063	0.034*	
C2	0.18591 (19)	0.3303 (4)	0.55824 (14)	0.0307 (5)	
H2A	0.1861	0.2514	0.6152	0.037*	
H2B	0.0987	0.3546	0.5353	0.037*	
C3	0.25190 (18)	0.5226 (3)	0.58044 (14)	0.0273 (4)	
H3A	0.2013	0.6016	0.6200	0.033*	
H3B	0.3328	0.4979	0.6152	0.033*	
C4	0.27179 (17)	0.6305 (3)	0.49340 (13)	0.0235 (4)	
C5	0.38514 (16)	0.5853 (3)	0.44868 (13)	0.0219 (4)	
H5	0.4400	0.4882	0.4824	0.026*	
C6	0.39582 (17)	0.5921 (3)	0.34653 (13)	0.0224 (4)	
H6	0.3146	0.6296	0.3137	0.027*	
C7	0.44268 (17)	0.4046 (3)	0.30577 (14)	0.0237 (4)	
H7	0.4961	0.3371	0.3552	0.028*	
C8	0.34519 (19)	0.2617 (3)	0.26488 (14)	0.0286 (4)	
H8A	0.2733	0.3358	0.2374	0.034*	
H8B	0.3807	0.1908	0.2140	0.034*	
C9	0.2983 (2)	0.1148 (3)	0.33238 (15)	0.0302 (5)	
H9A	0.2557	0.0098	0.2962	0.036*	
H9B	0.3705	0.0578	0.3688	0.036*	
C10	0.21163 (19)	0.1940 (3)	0.39806 (14)	0.0267 (4)	
C11	0.52534 (18)	0.4772 (3)	0.23293 (13)	0.0253 (4)	
H11	0.4763	0.4815	0.1714	0.030*	
C12	0.55874 (18)	0.6778 (3)	0.26267 (14)	0.0285 (5)	
C14	0.08422 (19)	0.2379 (4)	0.35502 (15)	0.0350 (5)	
H14A	0.0332	0.2884	0.4024	0.053*	
H14B	0.0465	0.1199	0.3285	0.053*	
H14C	0.0896	0.3340	0.3060	0.053*	
C15	0.15749 (18)	0.7084 (3)	0.44137 (15)	0.0297 (5)	
H15A	0.1226	0.8112	0.4776	0.045*	
H15B	0.0964	0.6050	0.4308	0.045*	
H15C	0.1787	0.7600	0.3817	0.045*	
C13	0.63833 (18)	0.3509 (4)	0.22548 (15)	0.0314 (5)	
H13A	0.6919	0.3579	0.2841	0.038*	
H13B	0.6116	0.2155	0.2161	0.038*	
N1	0.70913 (16)	0.4104 (3)	0.14878 (13)	0.0315 (4)	
H1N	0.715 (2)	0.545 (5)	0.1522 (19)	0.038*	
C2'	0.83004 (18)	0.3144 (4)	0.15110 (14)	0.0316 (5)	
H2'1	0.8667	0.3093	0.2159	0.038*	
H2'2	0.8861	0.3913	0.1149	0.038*	
C3'	0.82123 (19)	0.1106 (4)	0.11202 (15)	0.0340 (5)	
H3'1	0.7632	0.0347	0.1470	0.041*	
H3'2	0.7871	0.1160	0.0466	0.041*	
C4'	0.94557 (18)	0.0102 (4)	0.11730 (14)	0.0289 (5)	
C5'	0.9675 (2)	−0.1565 (4)	0.16744 (14)	0.0332 (5)	
H5'	0.9027	−0.2106	0.1995	0.040*	
C6'	1.0815 (2)	−0.2469 (4)	0.17225 (15)	0.0362 (5)	
H6'	1.0937	−0.3632	0.2063	0.043*	
C7'	1.17797 (19)	−0.1682 (4)	0.12744 (14)	0.0367 (6)	
C8'	1.15766 (19)	−0.0002 (4)	0.07758 (14)	0.0350 (5)	
H8'	1.2231	0.0558	0.0469	0.042*	
C9'	1.04241 (19)	0.0871 (4)	0.07215 (14)	0.0309 (5)	
H9'	1.0294	0.2017	0.0369	0.037*	
O1'	1.28929 (16)	−0.2601 (4)	0.13551 (11)	0.0526 (5)	
H1'	1.3325	−0.2226	0.0934	0.079*	
O1W1	0.5653 (4)	0.2900 (9)	−0.0060 (3)	0.0465 (12)	0.45
H1W1	0.5124	0.2239	0.0206	0.070*	0.45
H2W1	0.5295	0.3987	−0.0044	0.070*	0.45
O1W2	0.5563 (4)	0.4210 (10)	−0.0128 (3)	0.0465 (12)	0.40
H1W2	0.4812	0.3847	−0.0207	0.070*	0.40
H2W2	0.5479	0.5354	0.0065	0.070*	0.40
O1W3	0.5279 (11)	−0.0765 (18)	0.0245 (7)	0.036 (2)*	0.15
H1W3	0.4928	0.0289	0.0367	0.054*	0.15
H2W3	0.6044	−0.0506	0.0349	0.054*	0.15

### Atomic displacement parameters (Å^2^)

**Table:**

	*U*^11^	*U*^22^	*U*^33^	*U*^12^	*U*^13^	*U*^23^
O1	0.0240 (6)	0.0190 (8)	0.0332 (7)	−0.0028 (6)	0.0026 (5)	−0.0055 (6)
O2	0.0275 (7)	0.0209 (8)	0.0336 (7)	−0.0044 (6)	0.0079 (5)	−0.0018 (6)
O3	0.0432 (9)	0.0406 (11)	0.0417 (9)	−0.0141 (8)	0.0154 (7)	−0.0025 (8)
C1	0.0289 (9)	0.0174 (11)	0.0371 (11)	−0.0053 (8)	−0.0010 (8)	0.0026 (9)
C2	0.0308 (10)	0.0297 (12)	0.0313 (10)	−0.0076 (10)	0.0004 (8)	0.0020 (9)
C3	0.0248 (9)	0.0281 (13)	0.0289 (10)	−0.0036 (9)	0.0025 (7)	−0.0031 (9)
C4	0.0219 (9)	0.0194 (11)	0.0288 (9)	−0.0021 (8)	0.0001 (7)	−0.0048 (8)
C5	0.0204 (9)	0.0166 (10)	0.0282 (9)	−0.0009 (7)	−0.0016 (7)	−0.0018 (8)
C6	0.0180 (8)	0.0191 (11)	0.0301 (10)	−0.0009 (7)	0.0020 (7)	−0.0006 (8)
C7	0.0231 (9)	0.0204 (11)	0.0276 (9)	0.0025 (8)	0.0028 (7)	−0.0009 (8)
C8	0.0311 (10)	0.0238 (12)	0.0306 (10)	−0.0030 (9)	0.0012 (8)	−0.0054 (9)
C9	0.0355 (11)	0.0178 (12)	0.0369 (11)	−0.0033 (9)	0.0006 (8)	−0.0033 (9)
C10	0.0295 (10)	0.0176 (11)	0.0323 (10)	−0.0070 (8)	−0.0014 (8)	0.0018 (8)
C11	0.0243 (9)	0.0273 (12)	0.0240 (9)	0.0033 (8)	0.0000 (7)	−0.0008 (8)
C12	0.0253 (9)	0.0319 (13)	0.0283 (10)	−0.0023 (9)	0.0016 (8)	0.0032 (9)
C14	0.0293 (10)	0.0384 (14)	0.0367 (11)	−0.0051 (10)	−0.0019 (8)	−0.0033 (10)
C15	0.0207 (9)	0.0282 (12)	0.0403 (11)	0.0029 (8)	0.0031 (8)	−0.0022 (9)
C13	0.0268 (10)	0.0359 (13)	0.0316 (10)	0.0075 (9)	0.0034 (8)	0.0014 (9)
N1	0.0259 (9)	0.0344 (12)	0.0346 (9)	0.0058 (8)	0.0045 (7)	0.0057 (8)
C2'	0.0208 (9)	0.0407 (15)	0.0331 (10)	0.0041 (9)	0.0016 (8)	0.0003 (10)
C3'	0.0233 (10)	0.0441 (15)	0.0347 (10)	0.0005 (10)	0.0035 (8)	0.0018 (10)
C4'	0.0230 (10)	0.0358 (13)	0.0279 (10)	−0.0010 (9)	0.0030 (7)	−0.0053 (9)
C5'	0.0329 (10)	0.0375 (14)	0.0291 (10)	−0.0032 (10)	0.0014 (8)	−0.0015 (10)
C6'	0.0442 (12)	0.0331 (13)	0.0303 (10)	0.0037 (11)	−0.0025 (9)	−0.0003 (10)
C7'	0.0313 (10)	0.0530 (17)	0.0249 (9)	0.0134 (11)	−0.0037 (8)	−0.0071 (10)
C8'	0.0240 (10)	0.0523 (16)	0.0290 (10)	0.0037 (10)	0.0049 (8)	−0.0002 (10)
C9'	0.0260 (10)	0.0365 (14)	0.0308 (10)	0.0020 (9)	0.0051 (8)	0.0031 (10)
O1'	0.0398 (9)	0.0826 (15)	0.0345 (9)	0.0333 (10)	−0.0025 (7)	−0.0012 (9)
O1W1	0.0196 (11)	0.088 (4)	0.0315 (12)	−0.004 (2)	0.0016 (9)	−0.010 (3)
O1W2	0.0196 (11)	0.088 (4)	0.0315 (12)	−0.004 (2)	0.0016 (9)	−0.010 (3)

### Geometric parameters (Å, °)

**Table:**

O1—C5	1.444 (3)	C14—H14C	0.9800
O1—C4	1.445 (2)	C15—H15A	0.9800
O2—C12	1.339 (3)	C15—H15B	0.9800
O2—C6	1.465 (2)	C15—H15C	0.9800
O3—C12	1.199 (3)	C13—N1	1.460 (3)
C1—C10	1.330 (3)	C13—H13A	0.9900
C1—C2	1.494 (3)	C13—H13B	0.9900
C1—H1	0.9500	N1—C2'	1.468 (3)
C2—C3	1.537 (3)	N1—H1N	0.94 (3)
C2—H2A	0.9900	C2'—C3'	1.525 (4)
C2—H2B	0.9900	C2'—H2'1	0.9900
C3—C4	1.497 (3)	C2'—H2'2	0.9900
C3—H3A	0.9900	C3'—C4'	1.513 (3)
C3—H3B	0.9900	C3'—H3'1	0.9900
C4—C5	1.468 (3)	C3'—H3'2	0.9900
C4—C15	1.498 (3)	C4'—C5'	1.377 (4)
C5—C6	1.494 (3)	C4'—C9'	1.387 (3)
C5—H5	1.0000	C5'—C6'	1.382 (3)
C6—C7	1.533 (3)	C5'—H5'	0.9500
C6—H6	1.0000	C6'—C7'	1.386 (3)
C7—C11	1.524 (3)	C6'—H6'	0.9500
C7—C8	1.533 (3)	C7'—O1'	1.361 (3)
C7—H7	1.0000	C7'—C8'	1.381 (4)
C8—C9	1.528 (3)	C8'—C9'	1.384 (3)
C8—H8A	0.9900	C8'—H8'	0.9500
C8—H8B	0.9900	C9'—H9'	0.9500
C9—C10	1.494 (3)	O1'—H1'	0.8400
C9—H9A	0.9900	O1W1—H1W1	0.8500
C9—H9B	0.9900	O1W1—H2W1	0.8500
C10—C14	1.499 (3)	O1W1—H1W2	1.1302
C11—C12	1.494 (3)	O1W2—H2W1	0.3580
C11—C13	1.517 (3)	O1W2—H1W2	0.8500
C11—H11	1.0000	O1W2—H2W2	0.8500
C14—H14A	0.9800	O1W3—H1W3	0.8500
C14—H14B	0.9800	O1W3—H2W3	0.8500
			
C5—O1—C4	61.06 (12)	C7—C11—H11	109.1
C12—O2—C6	110.10 (16)	O3—C12—O2	121.2 (2)
C10—C1—C2	128.2 (2)	O3—C12—C11	127.7 (2)
C10—C1—H1	115.9	O2—C12—C11	111.07 (17)
C2—C1—H1	115.9	C10—C14—H14A	109.5
C1—C2—C3	109.84 (17)	C10—C14—H14B	109.5
C1—C2—H2A	109.7	H14A—C14—H14B	109.5
C3—C2—H2A	109.7	C10—C14—H14C	109.5
C1—C2—H2B	109.7	H14A—C14—H14C	109.5
C3—C2—H2B	109.7	H14B—C14—H14C	109.5
H2A—C2—H2B	108.2	C4—C15—H15A	109.5
C4—C3—C2	110.81 (16)	C4—C15—H15B	109.5
C4—C3—H3A	109.5	H15A—C15—H15B	109.5
C2—C3—H3A	109.5	C4—C15—H15C	109.5
C4—C3—H3B	109.5	H15A—C15—H15C	109.5
C2—C3—H3B	109.5	H15B—C15—H15C	109.5
H3A—C3—H3B	108.1	N1—C13—C11	111.50 (19)
O1—C4—C5	59.43 (12)	N1—C13—H13A	109.3
O1—C4—C3	116.76 (16)	C11—C13—H13A	109.3
C5—C4—C3	116.55 (18)	N1—C13—H13B	109.3
O1—C4—C15	112.73 (17)	C11—C13—H13B	109.3
C5—C4—C15	122.83 (17)	H13A—C13—H13B	108.0
C3—C4—C15	115.82 (17)	C13—N1—C2'	112.11 (18)
O1—C5—C4	59.51 (12)	C13—N1—H1N	106.4 (16)
O1—C5—C6	118.33 (17)	C2'—N1—H1N	113.1 (16)
C4—C5—C6	124.39 (16)	N1—C2'—C3'	112.61 (18)
O1—C5—H5	114.5	N1—C2'—H2'1	109.1
C4—C5—H5	114.5	C3'—C2'—H2'1	109.1
C6—C5—H5	114.5	N1—C2'—H2'2	109.1
O2—C6—C5	106.91 (15)	C3'—C2'—H2'2	109.1
O2—C6—C7	105.23 (14)	H2'1—C2'—H2'2	107.8
C5—C6—C7	114.15 (17)	C4'—C3'—C2'	112.36 (18)
O2—C6—H6	110.1	C4'—C3'—H3'1	109.1
C5—C6—H6	110.1	C2'—C3'—H3'1	109.1
C7—C6—H6	110.1	C4'—C3'—H3'2	109.1
C11—C7—C6	102.52 (17)	C2'—C3'—H3'2	109.1
C11—C7—C8	111.82 (16)	H3'1—C3'—H3'2	107.9
C6—C7—C8	117.38 (16)	C5'—C4'—C9'	117.9 (2)
C11—C7—H7	108.2	C5'—C4'—C3'	121.9 (2)
C6—C7—H7	108.2	C9'—C4'—C3'	120.2 (2)
C8—C7—H7	108.2	C4'—C5'—C6'	121.6 (2)
C9—C8—C7	116.07 (17)	C4'—C5'—H5'	119.2
C9—C8—H8A	108.3	C6'—C5'—H5'	119.2
C7—C8—H8A	108.3	C5'—C6'—C7'	120.1 (2)
C9—C8—H8B	108.3	C5'—C6'—H6'	120.0
C7—C8—H8B	108.3	C7'—C6'—H6'	120.0
H8A—C8—H8B	107.4	O1'—C7'—C8'	123.0 (2)
C10—C9—C8	114.82 (19)	O1'—C7'—C6'	118.1 (2)
C10—C9—H9A	108.6	C8'—C7'—C6'	118.9 (2)
C8—C9—H9A	108.6	C7'—C8'—C9'	120.3 (2)
C10—C9—H9B	108.6	C7'—C8'—H8'	119.8
C8—C9—H9B	108.6	C9'—C8'—H8'	119.8
H9A—C9—H9B	107.5	C8'—C9'—C4'	121.1 (2)
C1—C10—C9	120.28 (19)	C8'—C9'—H9'	119.4
C1—C10—C14	125.2 (2)	C4'—C9'—H9'	119.4
C9—C10—C14	114.49 (18)	C7'—O1'—H1'	109.5
C12—C11—C13	112.47 (17)	H1W1—O1W1—H2W1	98.2
C12—C11—C7	104.54 (17)	H1W1—O1W1—H1W2	80.6
C13—C11—C7	112.35 (18)	H2W1—O1W2—H2W2	100.5
C12—C11—H11	109.1	H1W2—O1W2—H2W2	101.3
C13—C11—H11	109.1	H1W3—O1W3—H2W3	103.2
			
C10—C1—C2—C3	−108.4 (2)	C8—C9—C10—C14	73.6 (2)
C1—C2—C3—C4	49.9 (2)	C6—C7—C11—C12	21.76 (18)
C5—O1—C4—C3	106.5 (2)	C8—C7—C11—C12	148.37 (17)
C5—O1—C4—C15	−115.80 (19)	C6—C7—C11—C13	144.01 (17)
C2—C3—C4—O1	−154.25 (17)	C8—C7—C11—C13	−89.4 (2)
C2—C3—C4—C5	−86.9 (2)	C6—O2—C12—O3	175.4 (2)
C2—C3—C4—C15	69.3 (2)	C6—O2—C12—C11	−5.9 (2)
C4—O1—C5—C6	115.29 (19)	C13—C11—C12—O3	45.5 (3)
C3—C4—C5—O1	−106.82 (19)	C7—C11—C12—O3	167.7 (2)
C15—C4—C5—O1	98.8 (2)	C13—C11—C12—O2	−133.00 (18)
O1—C4—C5—C6	−105.3 (2)	C7—C11—C12—O2	−10.8 (2)
C3—C4—C5—C6	147.85 (19)	C12—C11—C13—N1	−68.5 (2)
C15—C4—C5—C6	−6.5 (3)	C7—C11—C13—N1	173.84 (18)
C12—O2—C6—C5	141.89 (17)	C11—C13—N1—C2'	169.26 (19)
C12—O2—C6—C7	20.2 (2)	C13—N1—C2'—C3'	80.1 (2)
O1—C5—C6—O2	48.0 (2)	N1—C2'—C3'—C4'	−178.24 (18)
C4—C5—C6—O2	118.8 (2)	C2'—C3'—C4'—C5'	119.0 (2)
O1—C5—C6—C7	163.93 (15)	C2'—C3'—C4'—C9'	−59.9 (3)
C4—C5—C6—C7	−125.3 (2)	C9'—C4'—C5'—C6'	−0.8 (3)
O2—C6—C7—C11	−25.34 (18)	C3'—C4'—C5'—C6'	−179.7 (2)
C5—C6—C7—C11	−142.24 (16)	C4'—C5'—C6'—C7'	1.3 (3)
O2—C6—C7—C8	−148.29 (17)	C5'—C6'—C7'—O1'	178.7 (2)
C5—C6—C7—C8	94.8 (2)	C5'—C6'—C7'—C8'	−0.6 (3)
C11—C7—C8—C9	153.28 (18)	O1'—C7'—C8'—C9'	−179.8 (2)
C6—C7—C8—C9	−88.7 (2)	C6'—C7'—C8'—C9'	−0.4 (3)
C7—C8—C9—C10	74.3 (2)	C7'—C8'—C9'—C4'	0.9 (3)
C2—C1—C10—C9	169.1 (2)	C5'—C4'—C9'—C8'	−0.3 (3)
C2—C1—C10—C14	−9.4 (4)	C3'—C4'—C9'—C8'	178.7 (2)
C8—C9—C10—C1	−105.0 (2)		

### Hydrogen-bond geometry (Å, °)

**Table:**

*D*—H···*A*	*D*—H	H···*A*	*D*···*A*	*D*—H···*A*
N1—H1N···O3	0.94 (3)	2.22 (3)	2.979 (3)	137 (2)
O1'—H1'···O1W1^i^	0.84	1.75	2.568 (4)	164
O1'—H1'···O1W2^i^	0.84	2.01	2.832 (6)	166

Symmetry codes: (i) −*x*+2, *y*−1/2, −*z*.
